# LuxRep: a technical replicate-aware method for bisulfite sequencing data analysis

**DOI:** 10.1186/s12859-021-04546-1

**Published:** 2022-01-14

**Authors:** Maia H. Malonzo, Viivi Halla-aho, Mikko Konki, Riikka J. Lund, Harri Lähdesmäki

**Affiliations:** 1 Department of Computer Science, Aalto University, 00076 Espoo, Finland; 2 Turku Bioscience Centre, University of Turku and Åbo Akademi University, 20520 Turku, Finland

**Keywords:** Methylation, Bisulfite sequencing, Probabilistic

## Abstract

**Background:**

DNA methylation is commonly measured using bisulfite sequencing (BS-seq). The quality of a BS-seq library is measured by its bisulfite conversion efficiency. Libraries with low conversion rates are typically excluded from analysis resulting in reduced coverage and increased costs.

**Results:**

We have developed a probabilistic method and software, LuxRep, that implements a general linear model and simultaneously accounts for technical replicates (libraries from the same biological sample) from different bisulfite-converted DNA libraries. Using simulations and actual DNA methylation data, we show that including technical replicates with low bisulfite conversion rates generates more accurate estimates of methylation levels and differentially methylated sites. Moreover, using variational inference speeds up computation time necessary for whole genome analysis.

**Conclusions:**

In this work we show that taking into account technical replicates (i.e. libraries) of BS-seq data of varying bisulfite conversion rates, with their corresponding experimental parameters, improves methylation level estimation and differential methylation detection.

## Background

DNA methylation is a form of epigenetic regulation wherein cytosine is either methylated or demethylated. It is known to both repress and promote gene expression depending on its location relative to the target gene (e.g. CpG islands, shelves, shores or open sea) and pattern (hypomethylated or hypermethylated). As such, its dysregulation is associated with many diseases, including cancer. One of the most widely used methods for measuring DNA methylation is bisulfite sequencing [1]. When single-stranded DNA reacts with bisulfite, unmethylated cytosine is converted into uracil whereas methylated cytosine does not. Subsequent sequencing generates thymine in place of the converted unmethylated cytosine. To determine methylation counts, the resulting sequences are mapped to a reference genome to identify cytosine loci and so differentiate between unmethylated cytosine and thymine loci.

Several methods have been developed to estimate methylation levels and analyze differential methylation. One of the methods, Methylkit, uses two approaches, logistic regression (for samples with replicates) and Fisher’s exact test [2]. Another method, BSmooth, assumes that methylation count follows a binomial distribution and estimates methylation levels using a local likelihood smoother within a given window [3]. Many of the methods use the beta-binomial distribution to model methylation levels. RADmeth uses the beta-binomial regression model (with the logit link function) to estimate methylation levels [4]. BiSeq uses a binomial model in smoothing methylation levels within a window (cluster) with weights from a triangular kernel which is a function of distance between CpG loci [5]. MethylSig uses a beta-binomial approach with an approximation method for estimating the beta parameters [6]. MOABS, apart from using the beta-binomial model to estimate methylation levels, estimates a credible interval for the methylation difference between single cytosines (“credible methylation difference”) [7]. The paper mentions a feature for estimating bisulfite conversion rate but does not elaborate or mention if the estimate is integrated into the model estimating methylation. DSS-general also uses beta-binomial regression to model count data and it uses the arcsine link function [8]. DMRfinder clusters CpG sites into regions given a specified distance threshold then uses a hierarchical beta-binomial model [9]. Save for MOABS, none of these methods estimate bisulfite conversion rate and none, including MOABS, takes this rate into account when estimating methylation level or detecting differential methylation.

In the optimal case, the bisulfite conversion rate of a DNA library is high (e.g. above 99%) [10]. However, when an experiment yields a low conversion rate the common lab practice is to exclude the DNA library so as to avoid overestimation of methylation levels, resulting in additional costs or smaller sample size depending on whether a replacement library is prepared or not. An advanced computational approach to handle poor conversion rates would render exclusion of samples unnecessary. The methylation analysis method LuxGLM [11] estimates methylation levels from bisulfite sequencing data using a probabilistic model that accounts for bisulfite conversion rate. It showed that taking into account experimental parameters like bisulfite conversion efficiency improved accuracy of methylation analysis. However, though this model was able to handle biological replicates with a general linear model component, it assumed data from each sample consisted of only a single bisulfite-converted DNA library. In this work we propose LuxRep, an improved method and software to allow use of replicates from different DNA libraries with varying bisulfite conversion rates. To make LuxRep tool computationally efficient and thus more applicable to genome-wide analysis we also propose to use variational inference.

## Implementation

Our software consists of two modules: (1) estimation of experimental parameters from control data (“experimental parameters”) and (2) inference of methylation level (“biological parameters”) and differential methylation from DNA bisulfite sequencing data using the previously estimated experimental parameters. While LuxGLM was originally designed for analysis of both methylated (5mC) and hydroxymethylated cytosines (5hmC), the level for only one methylation modification (methylcytosine, 5mC) is included in this work (although our model can also be extended to 5hmC).

**Fig. 1:**
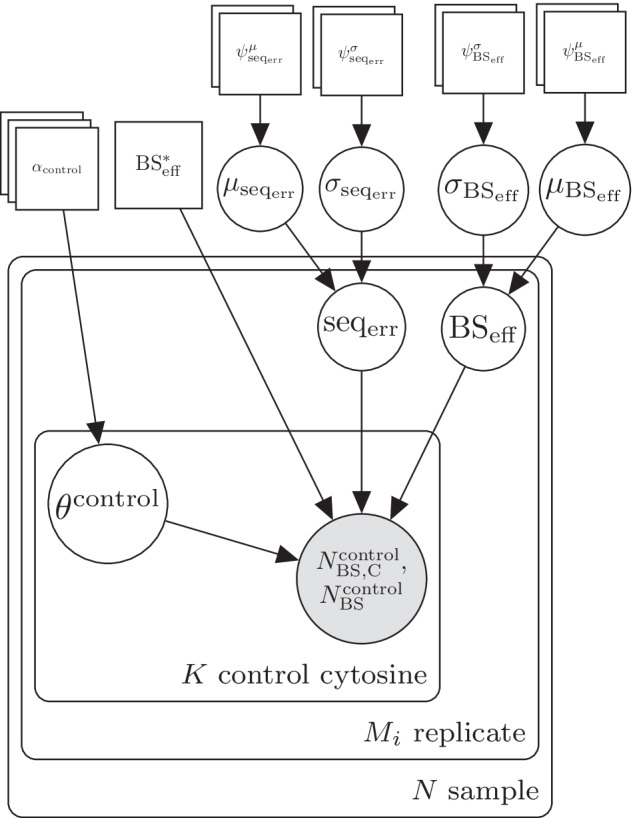
Plate diagram of LuxRep model for the module analyzing experimental parameters from control data

**Fig. 2:**
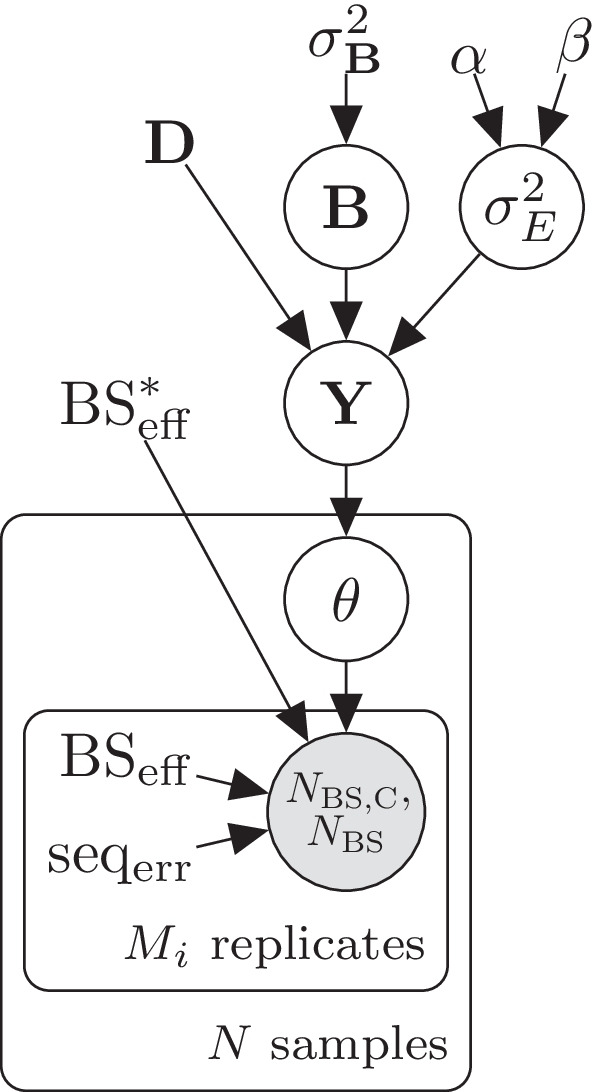
Plate diagram of the LuxRep model for estimating methylation level of a single cytosine with biological as well as technical replicates. The circles represent latent (white) and observed (gray) variables and the unbordered nodes represent constants

**Fig. 3:**
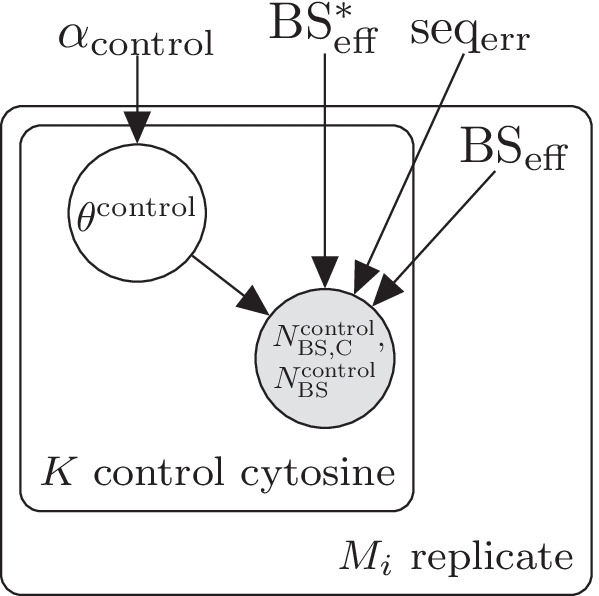
Plate diagram of LuxRep model for generating dummy control data

To facilitate genome wide analysis, in our model implementation the experimental parameters are first computed from the control data since all cytosines per technical replicate have the same value for these parameters (Fig. 1). Methylation levels are then determined individually for each cytosine, and differential methylation thereafter, using the pre-computed experimental parameters as fixed input (Fig. 2). We will next describe these two models in detail in Sects. 2.1–2.2.

### Experimental parameters

Methylation estimates are a function of experimental parameters: bisulfite conversion rate ($\text{BS}_{\rm eff}$), sequencing error ($\text{seq}_{\rm err}$) and incorrect bisulfite conversion rate ($\text{BS}^*_{\rm eff}$). A BS-seq library with low $\text{BS}_{\rm eff}$ results in overestimation of methylation levels. High $\text{seq}_{\rm err}$, on the other hand, can lead to both over and underestimation of methylation levels. Though typically not measured in high-throughput bisulfite sequencing experiments, high $\text{BS}^*_{\rm eff}$ leads to underestimation of methylation level.

To demonstrate that differences in technical parameters (specifically bisulfite conversion rate) is common we took a real bisulfite sequencing dataset [12] and compared the bisulfite conversion efficiencies of the technical replicates (i.e. libraries) per biological replicate (Additional file 1: Fig. S1). Most samples had significantly variable conversion rates, i.e. differences in technical parameters is common. Moreover, in practice, BS-seq datasets obtained with non-optimal conversion efficiencies are commonly ignored as currently there does not exist a statistical analysis tool that would allow analyzing BS-seq datasets with different conversion efficiencies. This in turn leads to loss of data, decrease in statistical power, loss of a biological sample, and increase in sequencing costs.

We start by briefly reviewing the underlying statistical model [11] and then introduce our extension that can handle technical replicates. Briefly, the conditional probability of a sequencing readout being “C” in BS-seq data is a function of the experimental parameters that include $\text{seq}_{\rm err}$ and $\text{BS}_{\rm eff}$, and depends on the methylation level $\theta \in [0,1]$. If a read was generated from an unmethylated cytosine (C), the conditional probability $p_{\rm{BS}}(\text{``C''|C})$ is given by

1 $$p_{\rm{BS}}(\text{``C''|C})=(1-\text{BS}_{\rm eff})(1-\text{seq}_{\rm err}) + \text{BS}_{\rm eff} \text{seq}_{\rm err}.$$

The term $(1-\text{BS}_{\rm eff})(1-\text{seq}_{\rm err})$ refers to the condition wherein unmethylated cytosine is incorrectly not converted into uracil and correctly sequenced as “C” whereas the term $\text{BS}_{\rm eff} \text{seq}_{\rm err}$ represents the condition wherein the unmethylated cytosine is correctly converted into uracil but incorrectly sequenced as “C”. Similarly, in the case of methylated cytosine

2 $$p_{\rm BS}(\text{``C''|5mC}) = (1-\text{BS}^*_{\rm eff})(1-\text{seq}_{\rm err}) + \text{BS}^*_{\rm eff} \text{seq}_{\rm err},$$

where $(1-\text{BS}^*_{\rm eff})(1-\text{seq}_{\rm err})$ denotes the case that methylated cytosine is correctly not converted to uracil and correctly sequenced as “C” while the term $\text{BS}^*_{\rm eff} \text{seq}_{\rm err}$ represents the case that methylated cytosine is incorrectly converted to uracil and incorrectly sequenced as “C”.

In [11], bisulfite conversion, sequencing error and incorrect bisulfite conversion rates were specific to each biological replicate, not technical replicate.

The experimental parameters follow a logistic normal distribution, where the bisulfite conversion rate $\text{BS}_{\rm eff}$ is given by

3 $$\text{BS}_{\rm eff}=\text{logit}^{-1}(\mu _{\rm{BS}_{\rm eff}} + \sigma _{\rm{BS}_{\rm eff}}r_{\rm{BS}_{\rm eff}})$$

and its hyperparameters are

4 $$\mu_{\rm{BS}_{\rm eff}} \sim {\mathcal {N}}(\psi ^{\mu ,\mu }_{\rm{BS}_{\rm eff}},\psi ^{\mu ,\sigma }_{\rm{BS}_{\rm eff}})$$

5 $$\text{ln}(\sigma _{{{\rm BS}_{\rm eff}}}) \sim {\mathcal {N}}(\psi ^{\sigma ,\mu }_{\rm{BS}_{\rm eff}},\psi ^{\sigma ,\sigma }_{\rm{BS}_{\rm eff}})$$

6 $$r_{\rm{BS}_{\rm eff}} \sim {\mathcal {N}}(0,1),$$

such that $\text{logit}(\text{BS}_{\rm eff}) \sim {\mathcal {N}}(\mu _{\rm{BS}_{\rm eff}},\sigma _{\rm{BS}_{\rm eff}})$, where $\mu _{\rm{BS}_{\rm eff}}$ is the mean and $\sigma _{\rm{BS}_{\rm eff}}$ is the standard deviation ($\psi ^{\mu ,\mu }_{\rm{BS}_{\rm eff}}=4$, $\psi ^{\mu ,\sigma }_{\rm{BS}_{\rm eff}}=1.29$, $\psi ^{\sigma ,\mu }_{\rm{BS}_{\rm eff}}=0.4$ and $\psi ^{\sigma ,\sigma }_{\rm{BS}_{\rm eff}}=0.5$). See [13] for details.

The sequencing error $\text{seq}_{\rm err}$ is modeled similarly

7 $$\text{seq}_{\rm err} = \text{logit}^{-1}(\mu _{\rm{seq}_{\rm err}} + \sigma _{\rm{seq}_{\rm err}}r_{\rm{seq}_{\rm err}})$$

8 $$\mu _{\rm{seq}_{\rm err}} \sim {\mathcal {N}}(\psi ^{\mu ,\mu }_{\rm{seq}_{\rm err}},\psi ^{\mu ,\sigma }_{\rm{seq}_{\rm err}})$$

9 $$\text{ln}(\sigma _{\rm{seq}_{\rm err}}) \sim {\mathcal {N}}(\psi ^{\sigma ,\mu }_{\rm{seq}_{\rm err}},\psi ^{\sigma ,\sigma }_{\rm{seq}_{\rm err}})$$

10 $$r_{\rm{seq}_{\rm err}} \sim {\mathcal {N}}(0,1),$$

such that $\text{logit}(\text{seq}_{\rm err}) \sim {\mathcal {N}}(\mu _{\rm{seq}_{\rm err}},\sigma _{\rm{seq}_{\rm err}})$, where $\mu _{\rm{seq}_{\rm err}}$ is the mean and $\sigma _{\rm{seq}_{\rm err}}$ is the standard deviation ($\psi ^{\mu ,\mu }_{\rm{seq}_{\rm err}}=-8$, $\psi ^{\mu ,\sigma }_{\rm{seq}_{\rm err}}=1.29$, $\psi ^{\sigma ,\mu }_{\rm{seq}_{\rm err}}=0.4$ and $\psi ^{\sigma ,\sigma }_{\rm{seq}_{\rm err}}=0.5$).

The hyperparameter values above were used since they worked well in a previously published related work [11] although we chose a lower $\psi ^{\mu ,\mu }_{\rm{seq}_{\rm err}}$ since it generated more robust methylation estimates with mid-values of theta (i.e. 0.3 and 0.7). Other than that, to confirm that the results were not sensitive to hyperparameter values we tested different values ranging from low ($\psi ^{\mu ,\mu }_{\rm{BS}_{\rm eff}}=1$, $\psi ^{\mu ,\sigma }_{\rm{BS}_{\rm eff}}=1$, $\psi ^{\sigma ,\mu }_{\rm{BS}_{\rm eff}}=0.1$, $\psi ^{\sigma ,\sigma }_{\rm{BS}_{\rm eff}}=0.1$, $\psi ^{\mu ,\mu }_{\rm{seq}_{\rm err}}=-10$, $\psi ^{\mu ,\sigma }_{\rm{seq}_{\rm err}}=1$, $\psi ^{\sigma ,\mu }_{\rm{seq}_{\rm err}}=0.1$ and $\psi ^{\sigma ,\sigma }_{\rm{seq}_{\rm err}}=0.1$) to high ($\psi ^{\mu ,\mu }_{\rm{BS}_{\rm eff}}=10$, $\psi ^{\mu ,\sigma }_{\rm{BS}_{\rm eff}}=10$, $\psi ^{\sigma ,\mu }_{\rm{BS}_{\rm eff}}=1$, $\psi ^{\sigma ,\sigma }_{\rm{BS}_{\rm eff}}=1$, $\psi ^{\mu ,\mu }_{\rm{seq}_{\rm err}}=-1$, $\psi ^{\mu ,\sigma }_{\rm{seq}_{\rm err}}=10$, $\psi ^{\sigma ,\mu }_{\rm{seq}_{\rm err}}=1$ and $\psi ^{\sigma ,\sigma }_{\rm{seq}_{\rm err}}=1$) hyperparameter values, relative to the values used in this paper, and indeed the methylation estimates were robust regardless of hyperparameter values (Additional file 1: Fig. S2).

The BS-seq experiments typically include completely unmethylated DNA fragments as controls (such as the lambda phage genome) that allow estimation of $\text{BS}_{\rm eff}$ and $\text{seq}_{\rm err}$. However, as BS-seq experiments typically do not include completely methylated DNA fragments as controls that would be needed to estimate the incorrect bisulfite conversion rate $\text{BS}^*_{\rm eff}$, it is set to a constant value (e.g. $\text{BS}^*_{\rm eff}=0$, see Sections "Estimating experimental parameters" and "Estimating methylation levels" for specific values used in results). Note also that the bisulfite conversion rate and sequencing error parameters are specific for each biological samples and technical replicate.

In Fig. 1, $\theta ^{\text {control}}$ represents the proportions of DNA methylation modifications in the control cytosine. In this case the proportion consists of unmethylated DNA, but this can be adjusted if additional DNA methylation modifications are included.

Following Eqs. 1 and 2, the observed total number of “C” readouts for a single control cytosine is binomially distributed,

11 $$N_{\rm{BS,C}}^{\text {control}} \sim \text {Bin}(N_\text{BS}^{\text {control}},p_{\rm{BS}}\text{(``C'')}^{\text {control}}),$$

where $N_{\rm{BS}}^{\text {control}}$ is the total number of reads and the probability of observing “C” is given by

12 $$\begin{aligned} p_{\rm{BS}}(\text{``C''})^{\text {control}}&= p_\text{BS}(\text{``C''} | \text{5mC})\theta ^{\text {control}} + p_\text{BS}(\text{``C''} | \text{C})(1-\theta ^{\text {control}}). \end{aligned}$$

Using the sequencing read counts from the control cytosines $N_{\rm{BS,C}}^{\text {control}}$ and $N_{\rm{BS}}^{\text {control}}$, posterior distributions of unknowns in this model are obtained using the inference methods described in section "Variational inference". Posterior means of $\text{BS}_{\rm eff}$ and $\text{seq}_{\rm err}$ (and $\text{BS}^*_{\rm eff}$ if available) are then used in the actual methylation level analysis as described in the next section.

### Biological parameters

For computing the biological parameters, the observed total number of “C” readouts for a single noncontrol cytosine is similar to Eq.  11, $N_{\rm{BS,C}} \sim \text {Bin}(N_\text{BS},p_{\rm{BS}}\text{(``C'')})$, where $N_{\rm{BS}}$ is the total number of reads and the probability of observing “C”, similar to Eq. 12, is given by

13 $$p_{\rm{BS}}(\text{``C''}) = p_\text{BS}(\text{``C''} | \text{5mC})\theta + p_\text{BS}(\text{``C''} | \text{C})(1-\theta )$$

where $$\theta =p(\text{5mC})$$.

LuxRep retains the general linear model with matrix normal distribution used by LuxGLM to handle covariates wherein matrix normal distribution is a generalisation of multivariate normal distribution to matrix-valued random variables. The following section summarizes the linear model (see [11] for more details).

In the general linear model component of LuxGLM (Fig. 2)

14 $${\mathbf {Y}}={{\mathbf {D}}}{{\mathbf {B}}} + {\mathbf {E}},$$

where ${\mathbf {Y}} \in {\mathbb {R}}^{N \times 2}$ contains the unnormalized methylation fractions, ${\mathbf {D}}$ is the design matrix (size *N*-by-*p*, where *p* is the number of parameters), ${\mathbf {B}} \in {\mathbb {R}}^{p \times 2}$ is the parameter matrix, and ${\mathbf {E}} \in {\mathbb {R}}^{N \times 2}$ is the noise matrix.

To derive the (normalized) methylation proportions $\varvec{\theta } = (\theta _1,\ldots ,\theta _N)^T$, LuxGLM uses the softmax link function (or transformation)

15 $$\theta _i = \text{Softmax}(\text{row}_i({\mathbf {Y}})).$$

The softmax function is obtained when generalizing the logistic function to multiple dimensions. That is, the softmax function $\sigma : {\mathbb {R}}^K \rightarrow [0,1]^K$ is defined by $\sigma ({\mathbf {z}})_i =$ $\frac{e^{z_i}}{ \sum _{j=1}^{K} e^{z_j} }$.

In matrix normal distribution,

16 $${\mathbf {X}} \sim {{\mathcal {M}}}{{\mathcal {N}}}({\mathbf {M}},{\mathbf {U}},{\mathbf {V}})$$

where ${\mathbf {M}}$ is the location matrix and ${\mathbf {U}}$ and ${\mathbf {V}}$ are scale matrices. Alternatively, ${\mathbf {X}}$ (in Eq.  16) can also be written as the multivariate normal distribution

17 $$\text{vec}({\mathbf {X}}) \sim {\mathcal {N}}(\text{vec}({\mathbf {M}}),{\mathbf {U}} \otimes {\mathbf {V}}),$$

where $\text{vec}(\cdot )$ denotes vectorization of a matrix and $\otimes$ denotes the Kronecker product.

Given Eq. 14, ${\mathbf {B}}$ and ${\mathbf {E}}$ take on the following prior distributions

18 $${\mathbf {E}}|\mathbf {U_E},\mathbf {V_E} \sim {{\mathcal {M}}}{{\mathcal {N}}}({\mathbf {0}}, \mathbf {U_E}, \mathbf {V_E})$$

19 $${\mathbf {B}}|\mathbf {M_B}, \mathbf {U_B}, \mathbf {V_B} \sim {{\mathcal {M}}}{{\mathcal {N}}}(\mathbf {M_B}, \mathbf {U_B}, \mathbf {V_B}).$$

Using the vectorized multivariate normal distribution formulation of the matrix normal distribution, matrix ${\mathbf {Y}}$ then becomes

20 $$\begin{aligned} &\text{vec}({\mathbf {Y}})|{\mathbf {D}}, {\mathbf {M_B}}, {\mathbf {U_B}}, {\mathbf {V_B}}, {\mathbf {U_E}}, {\mathbf {V_E}} \sim {\mathcal {N}}(({\mathbf {I}} \otimes {\mathbf {D}}) \text{vec} ({\mathbf {M_B}}), \\&({\mathbf {I}} \otimes {\mathbf {D}})({\mathbf {V_B}} \otimes {\mathbf {U_B}})({\mathbf {I}} \otimes {\mathbf {D}})^{\mathbf {T}} + {\mathbf {V_E}} \otimes {\mathbf {U_E}}). \end{aligned}$$

Assuming the scale matrices ${\mathbf {U_B}}$, ${\mathbf {V_B}}$, ${\mathbf {U_E}}$ and ${\mathbf {V_E}}$ are all diagonal with parameter and noise specific variances $\sigma ^\text{2}_{\mathbf {B}}$ and $\sigma ^\text{2}_{\mathbf {E}}$, probability densities for ${\mathbf {B}}$, ${\mathbf {E}}$ and ${\mathbf {Y}}$ can be stated as

21 $$\text{vec} ({\mathbf {B}}) \sim \ {\mathcal {N}}(\text{vec}({\mathbf {0}}), \sigma ^\text{2}_{\mathbf {B}}({\mathbf {I}} \otimes {\mathbf {I}}))$$

22 $$\text{vec} ({\mathbf {E}}) \sim \ {\mathcal {N}}(\text{vec}({\mathbf {0}}), \sigma ^\text{2}_{\mathbf {E}}({\mathbf {I}} \otimes {\mathbf {I}}))$$

23 $$\begin{aligned}&\text{vec}({\mathbf {Y}})|{\mathbf {D}},\sigma ^\text{2}_{\mathbf {B}}, \sigma ^\text{2}_{\mathbf {E}} \sim \ {\mathcal {N}}(\text{vec}({\mathbf {0}}), \nonumber \\&\sigma ^\text{2}_{\mathbf {B}} ({\mathbf {I}} \otimes {\mathbf {D}})({\mathbf {I}} \otimes {\mathbf {I}}) ({\mathbf {I}} \otimes {\mathbf {D}})^{\mathbf {T}} + \sigma ^\text{2}_{\mathbf {E}}({\mathbf {I}} \otimes {\mathbf {I}})). \end{aligned}$$

**Fig. 4:**
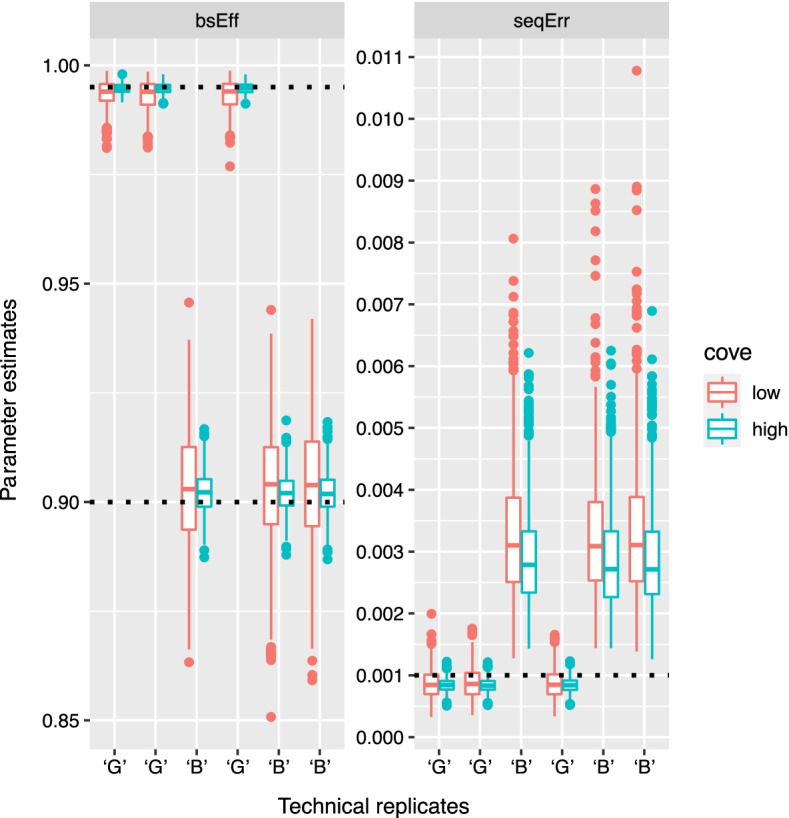
Parameter estimates of $\text{BS}_{\rm eff}$ and $\text{seq}_{\rm err}$. The x-axis shows whether the samples were drawn from ‘G’ (good quality) or ‘B’ (bad/low quality) technical replicates corresponding to $\text{BS}_{\rm eff}^B$ and $\text{BS}_{\rm eff}^G$, respectively, and grouped according to the two scenarios, discussed in section "Estimating methylation levels", ‘GGB’ and ‘GBB’. Low and high coverage refer to $N_{\rm{BS}} = 1 \dotsc 3$ and $N_{\rm{BS}} = 10$, respectively

**Fig. 5:**
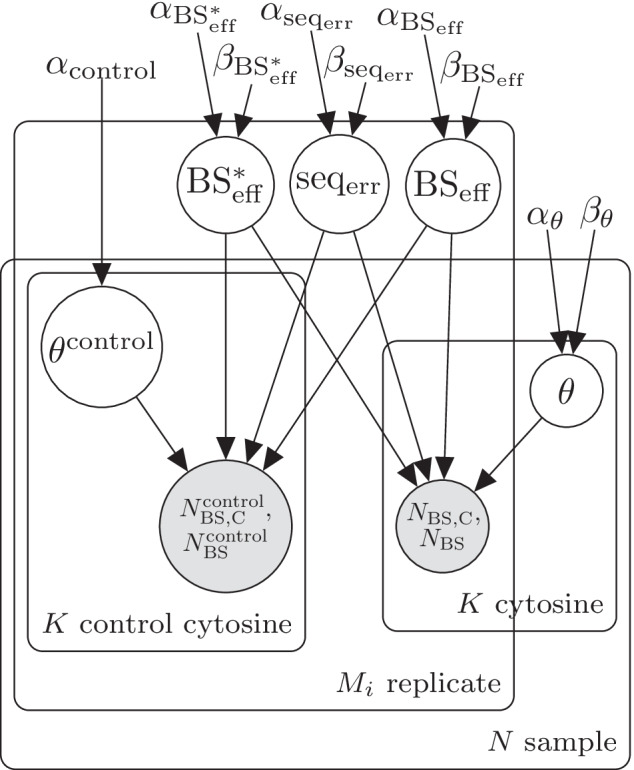
Plate diagram of LuxRep model for simulating data for estimating methylation level

**Fig. 6:**
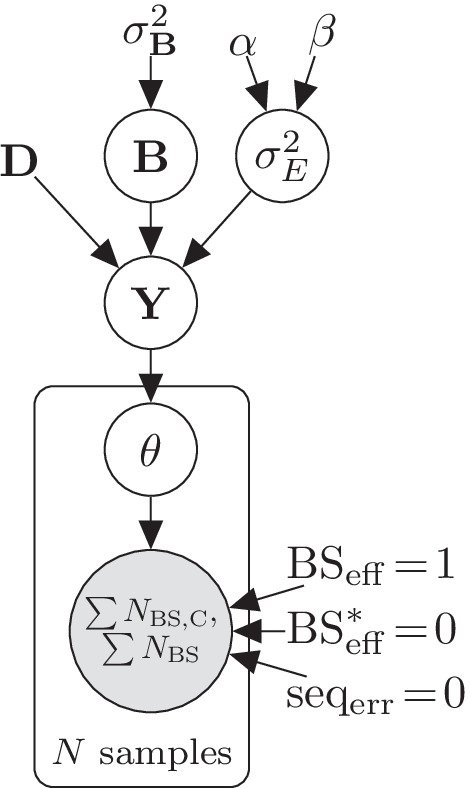
Plate diagram of the reduced LuxRep model that mimics the traditional approach of not accounting for experimental parameters

**Fig. 7:**
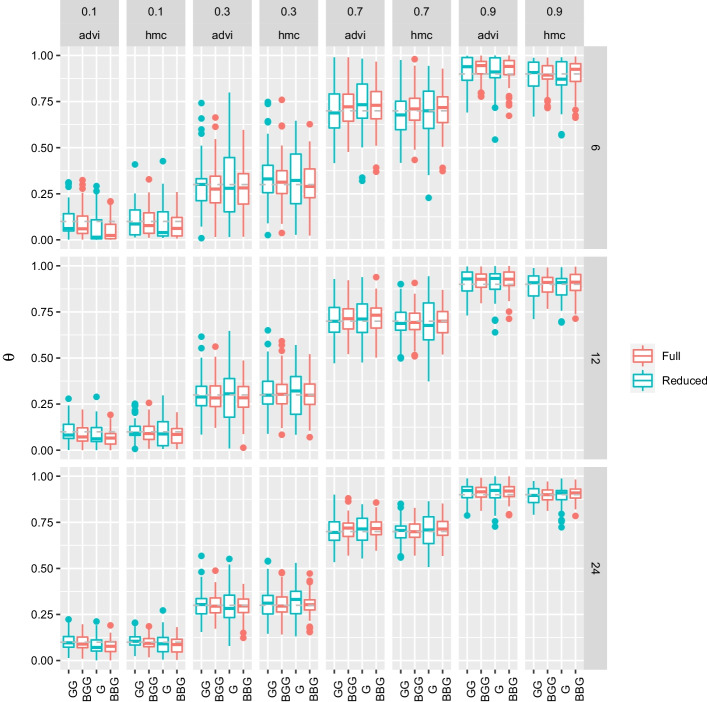
Boxplots of estimates of methylation levels. Datasets were analysed with the full and reduced LuxRep models with varying methylation levels (columns, values shown at topmost panel), varying number of reads (rows, values shown on right panel), different combinations of replicates with varying $\text{BS}_{\rm eff}$ (‘G’ and ‘B’) (x-axis), and using either HMC or ADVI to evaluate or approximate the posterior, respectively. The boxplots show the posterior means ($n=100$)

**Fig. 8:**
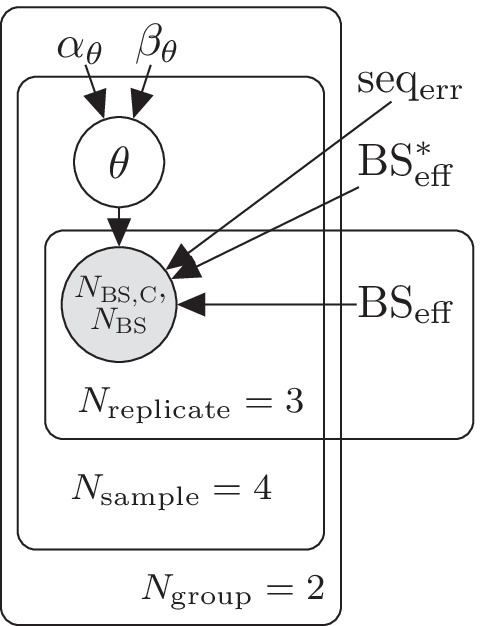
Plate diagram of model for generating dummy data for differential methylation analysis

**Fig. 9:**
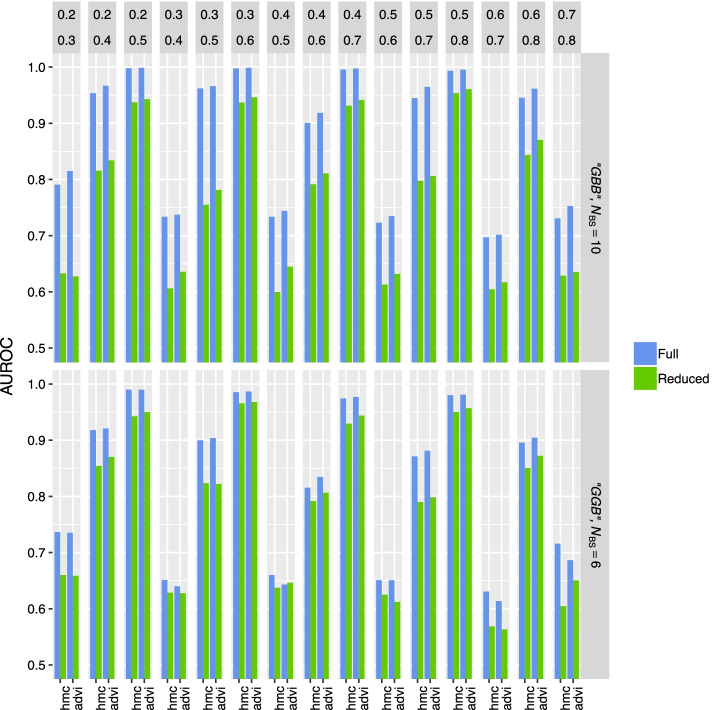
AUROCs of differential methylation calls. Accuracy in determining differential methylation was measured by generating datasets consisting of two groups (A and B) with varying $\Delta \theta$ ($\theta _A$ and $\theta _B$ levels are shown in top panels and when one or two of three replicates have low $\text{BS}_{\rm eff}$ (‘GGB’ and ‘GBB’, respectively.) For ‘GBB’ (top box) and ‘GGB’ (bottom box) $N_{\rm{BS}} = 10$ and $N_{\rm{BS}} = 6$, respectively, for each technical replicate. X-axis shows whether HMC or ADVI was used to evaluate or approximate the posteriors

Variance $\sigma ^2_{\mathbf {B}} = 5$ and $\sigma ^2_{\mathbf {E}} \sim \Gamma ^{-1}(\alpha ,\beta )$, where $\alpha = \beta = 1$, are used in this work. We chose to use the hyperparameter value $\sigma _B^2=5$ because that seems to be widely applicable and provides robust inference results. To confirm that the results are not sensitive to the particular choice of $\sigma _B^2$ value, we carried out an ablation study where we repeated the methylation level estimation experiment (from Fig. 7) with three different values of $\sigma _B^2$: 1, 5, and 10. Our results in Fig. S3 confirm that the results have very little or no variation depending on the choice of $\sigma _B^2$ value.

The inverse gamma distribution was used as prior for $\sigma _E^2$ since (with the alpha and beta hyperparameters used) it is uninformative and makes no strong assumptions with regards to the spread of the noise term. Also, the inverse gamma distribution is a conjugate prior to a normal distribution with known mean $\mu$ and unknown variance $\sigma ^2$.

We extend the model to allow modelling of technical replicates wherein the methylation level $\theta$ is the same for all different bisulfite-converted DNA libraries from the same biological sample but the experimental parameters ($\text{seq}_{\rm err}$ and $\text{BS}_{\rm eff}$) vary across both the biological replicates as well as the technical replicates.

In the modified model (Figs. 1 and 2), $N_{\rm{BS,C}}$ and $N_{\rm{BS}}$ represent the observed “C” and total counts, respectively, from each of the $M_i$ technical replicates per biological sample $i \in \left\{ 1,..,N\right\}$. Note that the experimental parameters $\text{BS}_{\rm eff}$ and $\text{seq}_{\rm err}$, taken from the posterior means, are sample and replicate-specific.

To detect differential methylation, hypothesis testing was done using Bayes factors (via the Savage-Dickey density ratio method) as implemented in [11].

### Variational inference

[11] used Hamiltonian Monte Carlo (HMC) for model inference (since the model is analytically intractable), whereas in variational inference (VI) the posterior $p(\phi |\mathbf{X} )$ of a model is approximated with a simpler distribution $q(\phi ;\rho )$, which is selected from a chosen family of distributions by minimizing Kullback-Leibler divergence between $p(\phi |\mathbf{X} )$ and $q(\phi ;\rho )$. We use the automatic differentiation variational inference algorithm (ADVI) from [14], which is integrated into Stan. ADVI is used to generate samples from the approximative posterior $q(\phi ;\rho )$.

There are a few parameters which can be tuned to make the ADVI algorithm [14] fast but accurate. These parameters are number samples used in Monte Carlo integration approximation of expectation lower bound (ELBO), number of samples used in Monte Carlo integration approximation of the gradients of the ELBO and number of samples taken from the approximative posterior distribution. The default values for gradient samples $N_G$ and ELBO samples $N_E$ are 100 and 1 respectively. Here we compare the computation times and preciseness of the Savage-Dickey estimate computed using HMC and ADVI with different $N_E$ and $N_G$ values. The tested values for $N_E$ were 100, 200, 500 and 1000 and for $N_G$ 1, 10 and 100. To make the HMC and ADVI methods comparable, the number of samples retrieved from the approximative posterior distribution is set to be the same for both methods.

To choose the best number of gradient samples and ELBO samples, simulation tests on LuxGLM model were executed. These tests were conducted in the following way: First, simulated data from the LuxGLM model was generated. The number of reads and replicates were varied (the tested values were 6, 12, 24 and 6, 10, 20 respectively) and for each combination data sets with differential methylation and without differential methylation were generated. The calculation of the Bayes factors was made using different $N_E$ and $N_G$ values. For each setting 100 data sets were simulated and Bayes factors were calculated. Using the computed Bayes factors, ROC curves and AUROC statistics were produced. Also, the computation times for each parameter value combination were taken down. The results of these tests for the case of 12 reads and 10 replicates are shown in Additional file 1: Figs. S4 and S5.

In Additional file 1: Fig. S4 the computation times for different parameter values are shown. In Additional file 1: Fig. S5 the computation time was plotted as a function of accuracy of the method when compared to the HMC approach. The average computation time for the HMC method is plotted in red. From the figures we can see that with the all tested parameter combinations the computing Savage-Dickey estimate with ADVI is faster than with HMC. In Additional file 1: Fig. S5, on the left side of the dashed line are the parameter combinations which gave better precision than HMC approach.

## Results

### Estimating experimental parameters

Samples prepared for BS-seq are typically spiked-in with unmethylated control DNA (often Lambda phage genome) that allows estimation of bisulfite conversion efficiency $\text{BS}_{\rm eff}$. For demonstration purposes, dummy control cytosine data were generated using the model illustrated in Fig. 3. Based on a cursory examination of an actual dataset generated from spiked-in Lambda phage DNA (data not shown), bisulfite sequencing data for 444 control cytosine were simulated with number of reads per cytosine $N_\text{BS} \in \{1, \dots ,3\}$. For comparison, another set-up was generated with coverage $N_\text{BS}=10$. Experimental parameters were set to fixed values while the methylation modification fractions $\theta ^{\text{control}}$ were drawn from $\text{Dir}(\alpha )$ (parameters listed below).

24 $$\begin{aligned} \alpha _\text{control}&= (999,1) \\ \text{BS}^*_{\rm eff}&= 0.001\\ \text{seq}_{\rm err}&= 0.001\\ \text{BS}_{\rm eff}&\in \{0.995,0.9\}\\ K_\text{control}&= 444\\ N_\text{BS}&\in \{1 \dots 3, 10\}\\ \\ \end{aligned}$$

The choice to use 90% as the low bisulfite conversion efficiency is based on Additional file 1: Fig. S1 which shows low conversion efficiencies to be around 90%. To test our method also with a lower conversion efficiency (<90%) we added the conversion efficiency 85% (Additional file 1: Fig. S6). As the plots show, the full model generates more accurate median on average than the reduced model also at 85% conversion efficiency.

Sequencing error and bisulfite conversion rates were estimated using the model illustrated in the plate diagram in Fig. 1 based on the dummy control cytosine data. Incorrect bisulfite conversion rate, $\text{BS}^*_{\rm eff}$, was set to a fixed value (0.1%) (in LuxGLM it was estimated from control data) because genome scale bisulfite sequencing typically do not include methylated cytosine control data. The data consists of *N* biological samples ($i \in \{1,\ldots ,N\}$), each of which has $M_i$ technical replicates corresponding to different bisulfite-converted DNA library preparations. The LuxGLM model [11] was modified to determine experimental parameters for each technical replicate separately (shown as the “replicates” plate in the diagram in Fig. 1). The circles represent latent (white) and observed (gray) variables and the squares/unbordered nodes represent fixed values (for parameters and hyperparameters).

Figure 4 shows the estimates for the experimental parameters. LuxRep generated good estimates for $\text{BS}_{\rm eff}$ and $\text{seq}_{\rm err}$, particularly with technical replicates that had high $\text{BS}_{\rm eff}$ (99.5%), even with extremely low coverage ($N_{BS}=1 \dotsc 3$). Technical replicates with higher coverage ($N_{BS}=10$), though, were more accurate in terms of median closer to the actual values and lower variance.

### Estimating methylation levels

For estimating methylation levels and analyzing differential methylation, we first simulated technical replicates with low ($\text{BS}_{\rm eff}^B \sim \text{beta}(90,10)$) and high ($\text{BS}_{\rm eff}^G \sim \text{beta}(99.5,0.5)$) BS conversion rates with varying sequencing depth $N_{\rm{BS}}$ and methylation level ($\theta \in [0.1,0.9]$). The datasets were generated following the model illustrated in Fig. 5 with methylation levels and experimental parameters generated following the beta distribution with parameters set to values listed below.

25 $$\begin{aligned}&\text{BS}_{\rm eff}^B \sim \text{beta}(90,10)\\&\text{BS}_{\rm eff}^G \sim \text{beta}(99.5,0.5)\\&\text{seq}_{\rm err} \sim \text{beta}(0.1,99.9)\\&\text{BS}^*_{\rm eff} \sim \text{beta}(0.1,99.9)\\&N_\text{BS} \in \{6,12,24\}\\&K_\text{cytosine}=4\\&\theta _1 \sim \text{beta}(100,900)\\&\theta _2 \sim \text{beta}(300,700)\\&\theta _3 \sim \text{beta}(700,300)\\&\theta _4 \sim \text{beta}(900,100)\\&N^\text{control}_\text{BS}=20\\&K^\text{control}_\text{cytosine}=100\\&\theta ^\text{control} \sim \text{Dir}(999,1)\\ \end{aligned}$$

where the Dirichlet distribution is denoted by Dir($\cdot$).

Two scenarios were simulated consisting of three technical replicates each: (i) two replicates with high $\text{BS}_{\rm eff}$ (i.e. good samples, ‘G’) and one with low $\text{BS}_{\rm eff}$ (i.e. bad sample, ‘B’)(‘GGB’), and (ii) one ‘G’ replicate and two ‘B’ replicates (‘GBB’). Each scenario was analyzed using (i) the *full* LuxRep model (Fig. 2) and (ii) a *reduced* model with experimental parameters fixed to $\text{BS}_{\rm eff}=1$, $\text{seq}_{\rm err}=0$ and $\text{BS}^*_{\rm eff}=0$, and using the “C” and “T” counts from only the ‘G’ samples (those with $\text{BS}_{\rm eff}=99.5\%$ and above) to simulate the traditional approach of not accounting for experimental parameters (Fig. 6). Results from estimating the models with HMC and ADVI were also compared.

Datasets ($n=100$) were analysed with the full and reduced LuxRep models with varying methylation levels, varying number of reads, different combinations of replicates with varying $\text{BS}_{\rm eff}$ (‘G’ and ‘B’), and using either HMC or ADVI to evaluate or approximate the posterior, respectively (Fig. 7). For each simulated data set we estimated the methylation level $\theta$ using the posterior mean of samples ($S=1000$) drawn from the posterior (HMC) and approximate posterior (ADVI) distribution.

The variance of the estimates using the full model was generally lower compared to the reduced model across $\theta$ and $N_{\rm {BS}}$ values (Fig. 7) demonstrating the utility of using LuxRep with replicates of varying $\text{BS}_{\rm eff}$. The decrease in variance was generally greater with the second scenario (‘GBB’), highlighting the capability of LuxRep to make use of samples with low $\text{BS}_{\rm eff}$. Improvements in the estimates were comparable when using HMC and ADVI. Notable also is the comparable accuracy between the two scenarios ‘GGB’ and ‘GBB’, i.e. ‘GBB’ was relatively as accurate as ‘GGB’ even though it had more replicates with low $\text{BS}_{\rm eff}$.

To more directly address the question of whether the full model significantly improves accuracy compared with traditional methods we performed methylation estimation using the full and reduced (representing traditional methods) methods with varying bisulfite conversion rates, including all samples for both the full and reduced models (Additional file 1: Fig. S6). Lower bisulfite conversion rates (85% and 90%) generated greater differences in estimates with the full model generally showing a more accurate median, specially with $\theta$ values of 0.3 and 0.7. The median were generally similar with higher bisulfite conversion rates. In terms of variance, the differences varied according to methylation level and bisulfite conversion rate (e.g. the variance of the full model was generally slightly higher with $\theta$ values of 0.1 and 0.3, whereas the variance of the reduced model was generally higher with theta 0.9).

Since most genomic regions tend to be unmethylated we queried the estimates when the actual methylation level approaches zero ($\theta = 0.1$). As shown in Fig. 7 and Additional file 1: Fig. S6, at low methylation levels (e.g. 0.1), the median is below the actual value, that is the methylation levels tend to be underestimated. It follows that for genomic regions that are unmethylated it is unlikely that the method will erroneously estimate a higher methylation level.

To test the utility of LuxRep on an actual bisulfite sequencing dataset, methylation levels were estimated from an RRBS dataset [12] consisting of two individuals and three replicates each (two low and one high $\text{BS}_{\rm eff}$, individual 1: 96.38%, 99.32% and 99.96%; individual 2: 94.59%, 98.67% and 99.98%). The replicate with high $\text{BS}_{\rm eff}$ was analyzed with the full model while the two low $\text{BS}_{\rm eff}$ replicates were analyzed with both the full and reduced models. The difference in the estimated methylation levels (1000 CpG sites) between the high $\text{BS}_{\rm eff}$ replicate and the low $\text{BS}_{\rm eff}$ replicates using the full and reduced models were measured by taking their Euclidean distance which showed greater similarity when using the full model (individual 1: reduced: 2.29, full: 2.23; individual 2: reduced: 2.55, full: 2.49).

### Detecting differential methylation

Accuracy in determining differential methylation was measured by generating datasets consisting of two groups (A and B) with varying methylation level difference $\Delta \theta$ between the two groups and when one or two of three replicates have low $\text{BS}_{\rm eff}$ (‘GGB’ and ‘GBB’, respectively). Each group consisted of four biological replicates wherein each biological replicate had three technical replicates each (with different sequencing read coverage, $N_{\rm{BS}}=10$ or $N_{\rm{BS}}=6$; the standard threshold for total sequencing read coverage is $N_{\rm{BS}}=10$). The model for generating simulated data is described in Fig. 8 (where $\theta \sim \text{Beta}(\alpha _\theta ,\beta _\theta )$, with parameters shown in Table 1).

**Table 1:** $${\overline{\theta }}$$ parameters

${\overline{\theta }}$	$\alpha _\theta$	$\beta _\theta$
0.2	200	800
0.3	300	700
0.4	400	600
0.5	500	500
0.6	600	400
0.7	700	300
0.8	800	200

Differential methylation was analysed using the full and reduced LuxRep models (see Figs. 2 and 6, respectively, and, for additional details of hyperpriors used, [11]), evaluated with HMC and ADVI. Eq. 26 shows the design matrix ${\mathbf {D}}$ and parameter matrix ${\mathbf {B}}$ used in the general linear model component (Bayes factors were computed using the Savage-Dickey density ratio estimator using samples of $b_\text{2,1}$ and $b_\text{2,2}$, $S=1600$ and $S=1000$ from the posterior distributions approximated with HMC and ADVI, respectively).26AUROCs were calculated based on $\sim$ $200$ positive ($\Delta \theta \ne 0$) and $\sim$ $200$ negative ($\Delta \theta =0$) samples (Fig. 9). The full model consistently generated higher AUROCs compared to the reduced model, moreso with the ‘GBB’ subsets, showing that LuxRep is able to utilize DNA libraries with low $\text{BS}_{\rm eff}$ to improve differential methylation analysis.

**Fig. 10:**
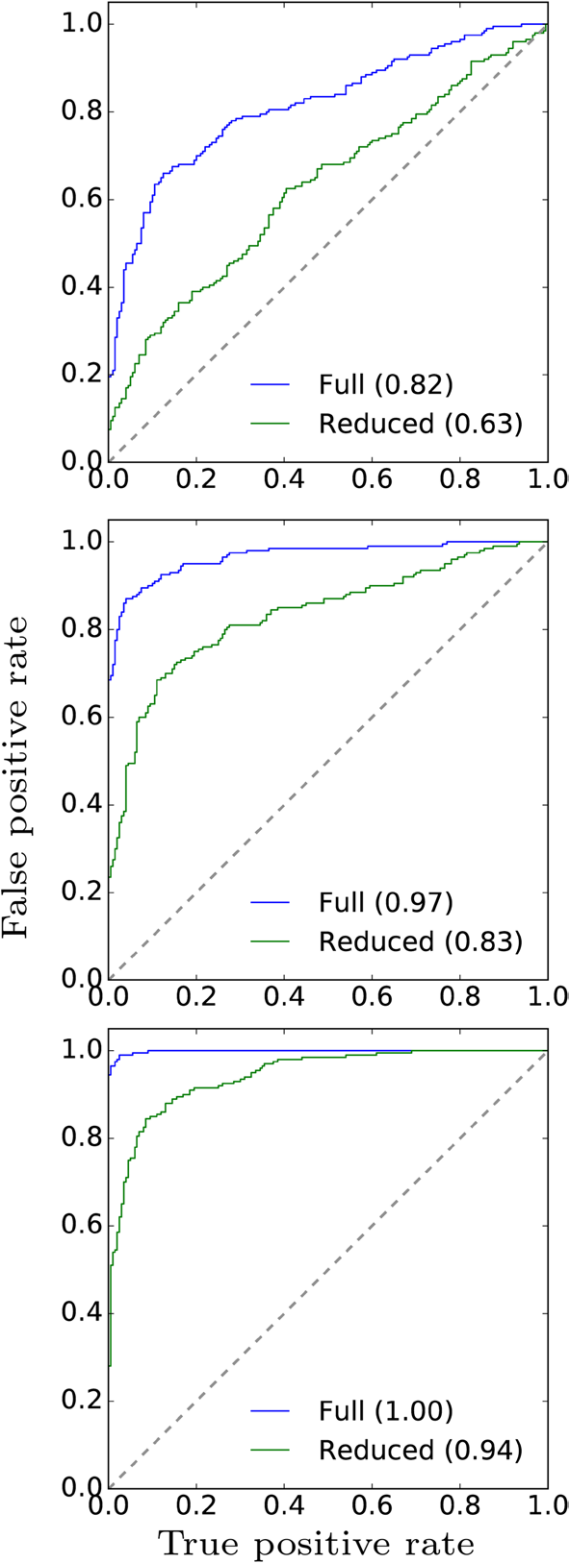
Select ROC curves of differential methylation calls generated from the full and reduced models (with technical replicates ‘GBB’ and ‘G’, respectively) where $\theta _\text{A}=0.2$ and $\theta _\text{B}$ was set to 0.3, 0.4 and 0.5 (top, middle and bottom panels, respectively). Samples were generated from the approximated posterior using variational inference

**Fig. 11:**
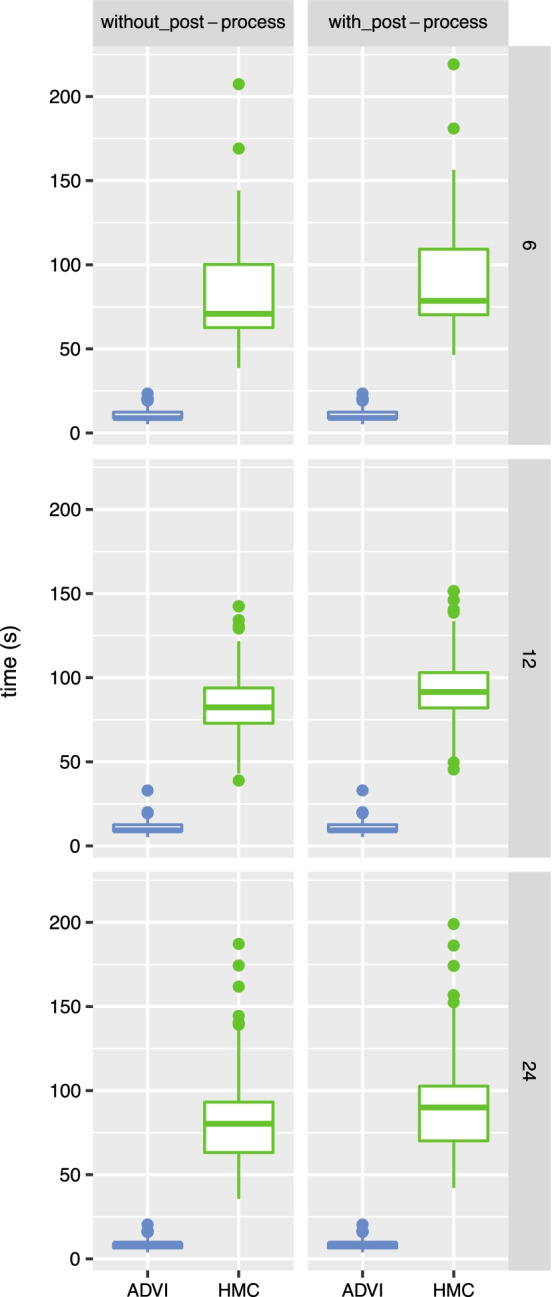
Comparison of running times using HMC and ADVI for model evaluation

Select ROC curves generated from the full and reduced models show notable increase in AUROCs when using the full over the reduced model (Fig. 10). Moreover, the difference in AUROCs increases with decreasing $\Delta \theta$.

In addition to AUROC, to provide empirical statistical power, we calculated the true positive rates for differential methylation (Additional file 1: Fig. S7). True positive rates were generally higher in the full model compared to the reduced model, as expected.

### Comparing running times

Running times were measured using the Stan [15] time records and by a Python function, and with or without the additional time required for post-processing the output files (i.e. parsing relevant information), with varying number of reads (Fig.11). The computations were performed using a computing cluster; a single core with 2GB memory was used for ADVI approximation (HMC sampling could be more efficiently run with one core for each MCMC chain hence run time was based on the slowest chain). Significant reduction in running times were observed with using ADVI over HMC.

## Conclusions

LuxRep tool described in this paper allows technical replicates with varying bisulfite conversion efficiency to be included in the analysis. LuxRep improves the accuracy of methylation level estimates and differential methylation analysis and lowers running time of model-based DNA methylation analysis by using ADVI.

## Supplementary Information

**Additional file 1:** Additional figures containing: (**i**) Demonstrating differences in technical parameters, (**ii**) Testing different hyperparameters for sequencing error and bisulfite conversion rates, (**iii**) Testing different $\sigma_B^2$ for methylation level estimation, (**iv**) Choosing parameters for variational inference, (**v**) Comparing full and reduced models in methylation level estimation, and (**vi**) True positive rates of differential methylation.

## Data Availability

LuxRep is open source and freely available from https://github.com/tare/LuxGLM/tree/master/LuxRep. Datasets that support the findings of this study are available in [12].

## Abbreviations

ADVI: Automatic differentiation variational inference
BS-seq: Bisulfite sequencing

## Declarations

### Availability and requirements

Project name: LuxRep. Project home page: https://github.com/tare/LuxGLM/tree/master/LuxRep. Operating system(s): Mac OSX and Linux. Programming language: Python, CmdStan, pystan, Numpy, Scipy. Other requirements: CmdStan (tested on version 2.18.0), Python (tested on version 3.7.5), pystan (tested on version 2.17.1.0), Numpy (tested on version 1.20.2), Scipy (tested on version 1.1.0). LuxRep is freely available at https://github.com/tare/LuxGLM/tree/master/LuxRep along with documentation. License: MIT License Any restrictions to use by non-academics: Not applicable.

## References

[1] Frommer M, McDonald LE, Millar DS, Collis CM, Watt F, Grigg GW, Molloy PL, Paul CL (1992). A genomic sequencing protocol that yields a positive display of 5-methylcytosine residues in individual dna strand. Nucleic acids research. Proc Natl Acad Sci USA.

[2] Akalin A, Kormaksson M, Li S (2012). methylkit: a comprehensive r package for the analysis of genome-wide dna methylation profiles. Genome Biol.

[3] Hansen KD, B L, Irizarry RA. Bsmooth: from whole genome bisulfite sequencing reads to differentially methylated regions. Genome biology 2012; 3, 1–10. 10.1186/gb-2012-13-10-r83 PMC3491411 23034175

[4] Dolzhenko E, Smith AD (2014). Using beta-binomial regression for high-precision differential methylation analysis in multifactor whole-genome bisulfite sequencing experiments. BMC Bioinform.

[5] Hebestreit K, Dugas M, Hans-Ulrich K (2013). Detection of significantly differentially methylated regions in targeted bisulfite sequencing data. Bioinformatics.

[6] Park Y, Figueroa ME, Rozek LS, Sartor MA (2014). Methylsig: a whole genome dna methylation analysis pipeline. Bioinformatics.

[7] Sun D, Xi Y, Rodriguez B, Park HJ, Tong P, Meong M, Goodell MA, Li W (2014). Moabs: model based analysis of bisulfite sequencing data. Genome Biol.

[8] Park Y, Hao W (2016). Differential methylation analysis for bs-seq data under general experimental design. Bioinformatics.

[9] Gaspar JM, Hart PH (2017). Dmrfinder: efficiently identifying differentially methylated regions from methylc-seq data. BMC Bioinform.

[10] Wreczycka K, Gosdschan A, Yusuf D, Grüning B, Assenov Y, Akalin A (2017). Strategies for analyzing bisulfite sequencing data. J Biotechnol.

[11] Äijö T, Huang Y, Mannerström H, Chavez L, Tsagaratou A, Rao A, Lähdesmäki H (2016). A probabilistic generative model for quantification of dna modifications enables analysis of demethylation pathways. Genome Biol.

[12] Konki M, Malonzo M, Karlsson IK, Lindgren N, Ghimire B, Smolander J, Scheinin NM, Ollikainen M, Laiho A, Elo LL, Lönnberg T, Matias R, Pedersen NL, Kaprio J, Lähdesmäki H, Rinne JO, Lund RJ (2019). Peripheral blood dna methylation differences in twin pairs discordant for alzheimer’s disease. Clin Epigenet.

[13] Äijö T, Yue X, Rao A, Lähdesmäki H (2016). Luxglm: a probabilistic covariate model for quantification of dna methylation modifications with complex experimental design. Bioinformatics.

[14] Kucukelbir A, Ranganath R, Gelman A, Blei D. Automatic variational inference in stan. In: Cortes, C, Lee DD, Sugiyama M, R G (eds) Advances in neural information processing systems 28 (NIPS 2015), pp. 568–576 2015. Neural Information Processing Systems.

[15] Carpenter B, Gelman A, Hoffman MD, Lee D, Goodrich B, Betancourt MB, Guo J, Li P, Riddell A (2017). Stan: a probabilistic programming language. J Stat Software.

