# Supplementary material for: LuxRep: a technical replicate-aware method for bisulfite sequencing data analysis
Source: BMC Bioinformatics. 2022 Jan 14;23:41. doi: 10.1186/s12859-021-04546-1 (PMC8760685; doi:10.1186/s12859-021-04546-1)
Supplement: Supplementary file 1 — Additional file 1: Additional figures containing: (i) Demonstrating differences in technical parameters, (ii) Testing different hyperparameters for sequencing error and bisulfite conversion rates, (iii) Testing different $\sigma_B^2$σB2 for methylation level estimation, (iv) Choosing parameters for variational inference, (v) Comparing full and reduced models in methylation level estimation, and (vi) True positive rates of differential methylation. [file 12859_2021_4546_MOESM1_ESM.pdf]

LuxRep: a technical replicate-aware method for bisulfite sequencing data analysis

Supplementary information

Maia Malonzo, Viivi Halla-aho, Mikko Konki, Riikka Lund,
Harri Lähdesmäki

December 9, 2021

1 Demonstrating differences in technical parameters

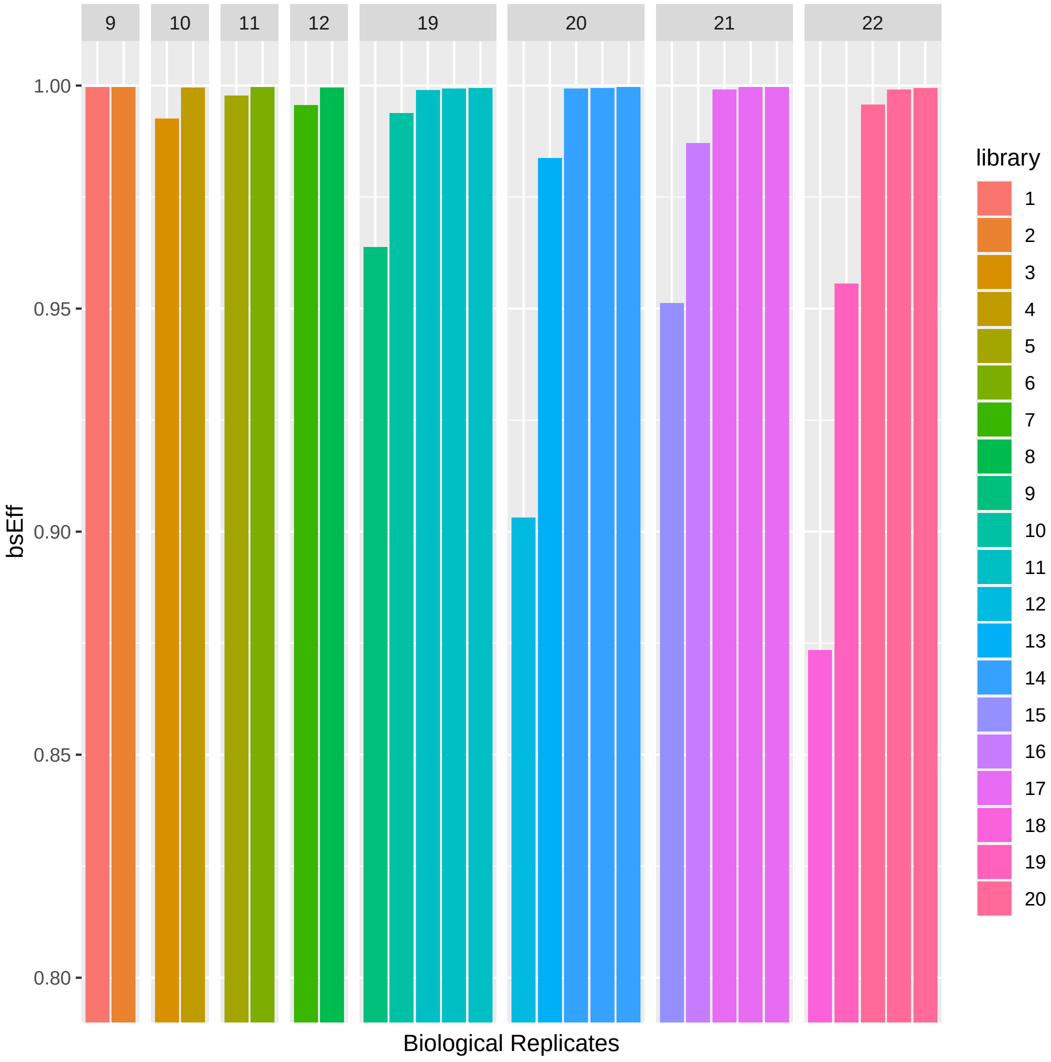

Figure S1: The barplots show the bisulfite conversion rates for different libraries taken from a real bisulfite sequencing dataset. The number of libraries ranged from two to three for each of eight biological replicates. On the top x-axis are indices of biological replicates and the colors represent different libraries for each biological replicate.

2 Testing different hyperparameters for sequencing error and bisulfite conversion rates

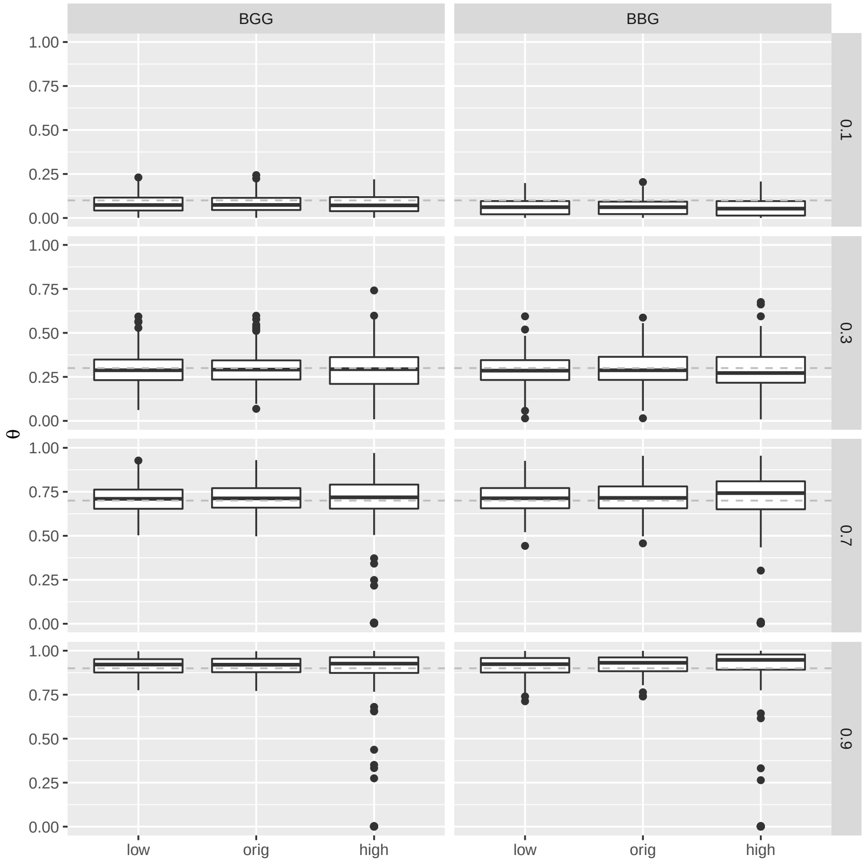

Figure S2: The boxplots compare θ estimates with varying hyperparameter values (see text in manuscript). We used both lower and higher hyperparameter values (bottom x-axis) relative to the ones originally used in this paper ('orig'). On the top x-axis are the different combination of technical replicates with varying bisulfite conversion rates. On the right y-axis are the actual methylation levels (and grey dashed lines).

3 Testing different σ_B^2 for methylation level estimation

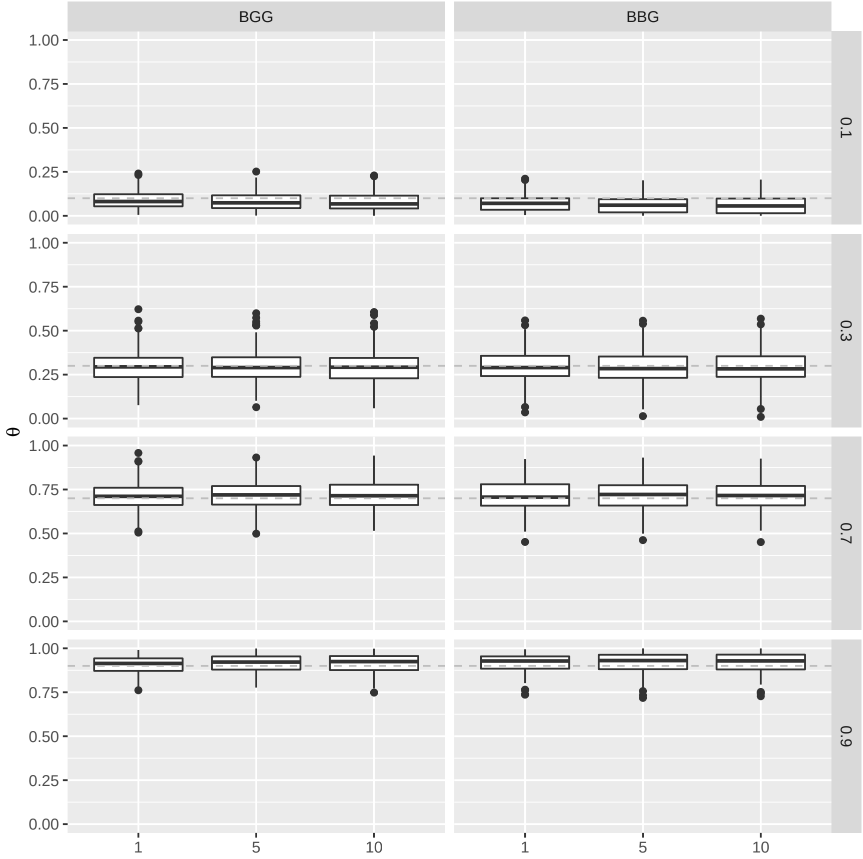

Figure S3: The boxplots visualize theta estimates (200 samples each) with varying values of σ_B^2. On the top panel BGG and BBG represent the composition of bisulfite conversion rates: B - 0.9 and G - 0.995. We analyzed 4 biological replicates with average methylation levels θ=0.1, 0.3, 0.7 and 0.9, with 3 technical replicates each (either BGG or BBG). On the bottom x-axis are the values used for σ_B^2 (the value used in the paper is 5). On the left y-axis are θ values and on the right are the actual θ values (also represented by dashed lines). The theta estimates across σ_B^2 values are similar, indicating that the results are not sensitive to the choice of σ_B^2 hyperparameter value.

4 Choosing parameters for variational inference

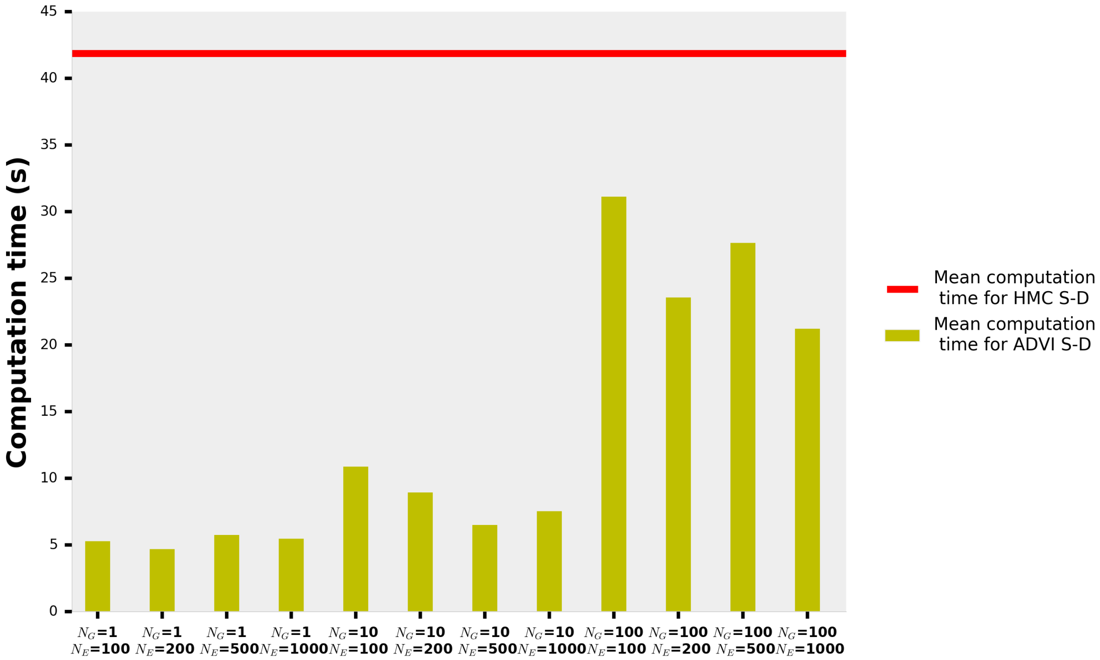

Figure S4: A barplot of the computation times for ADVI with different parameters N_E and N_G. The red line shows the mean computation time with HMC sampler.

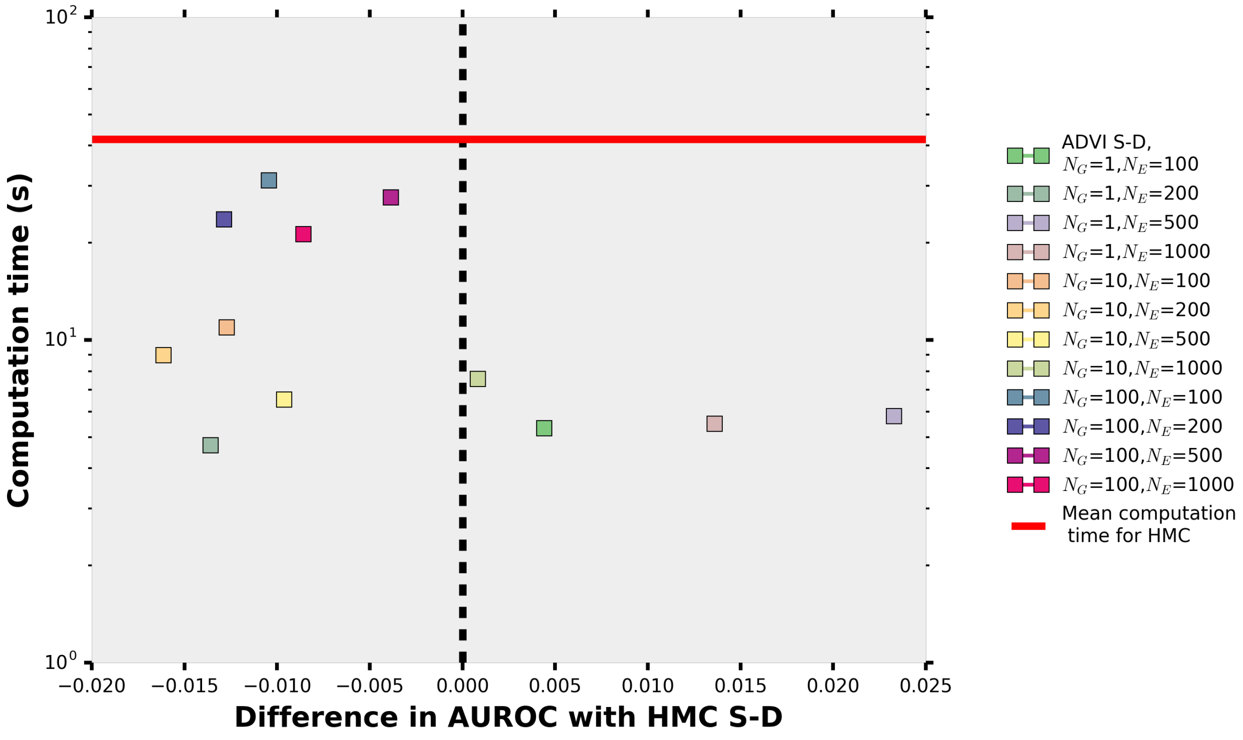

Figure S5: A scatterplot of the computation times with ADVI as function of difference of AUROC values. The different parameter parameter values N_E and N_G . The red line shows the mean computation time for HMC sampler. The dots on the left side of the black dotted line show higher accuracy than Savage-Dickey calculated with HMC and the dots on the right side show lower accuracy than HMC Savage-Dickey. The computation times are plotted in logarithmic scale, and it can be seen that the computation times for ADVI are one magnitude smaller than for HMC when using a good choice of parameters. And as the graph suggests, even when the computations are done considerably faster than with HMC, ADVI is almost as precise as HMC or even more precise.

5 Comparing full and reduced models in methylation level estimation

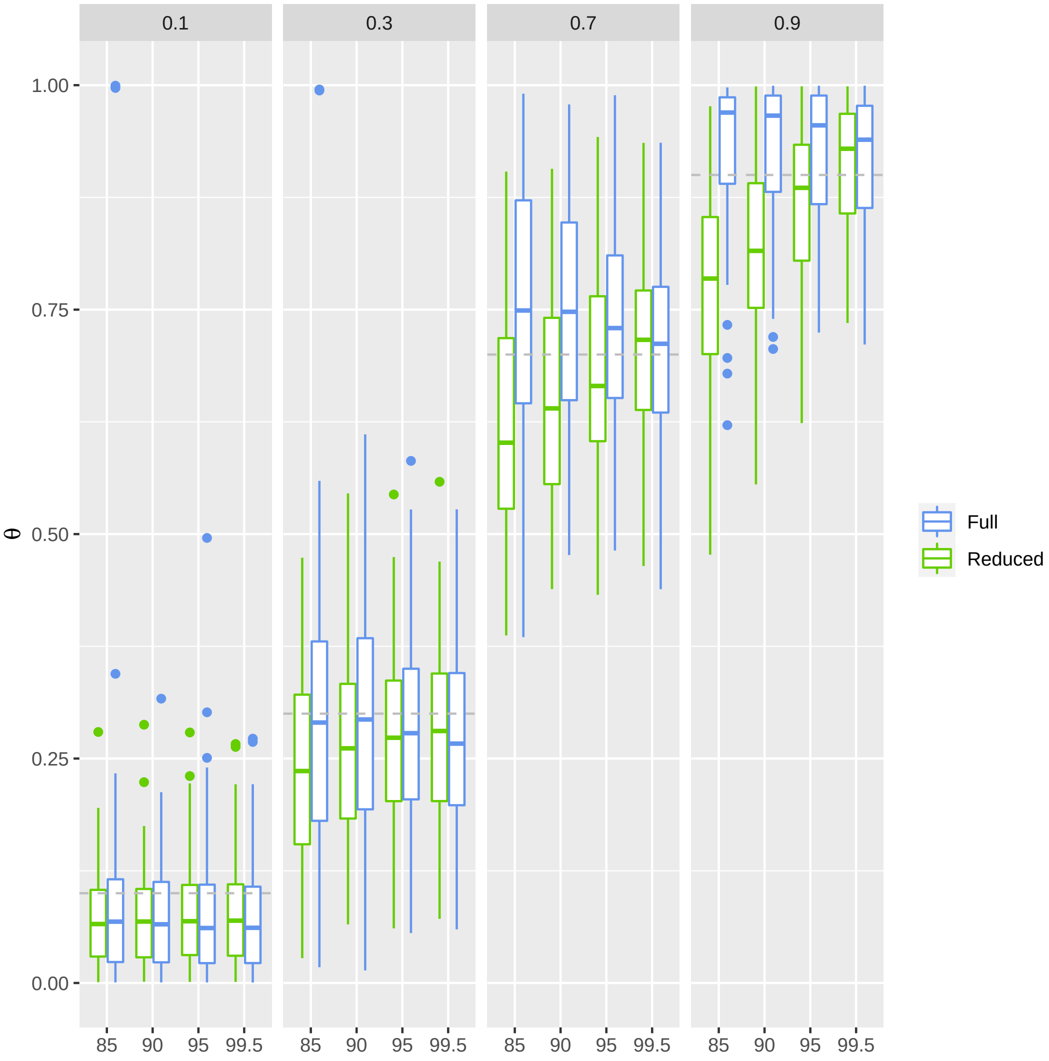

Figure S6: The boxplots compare theta estimates generated from varying bisulfite conversion rates when using the full and reduced models. For the full model two technical replicates were used whereas with the reduced model the reads between the two technical replicates were summed up. The top x-axis shows the actual methylation level (also in grey dashed lines) and the bottom x-axis shows the bisulfite conversion rates. Each boxplot consisted of 100 samples.

6 True positive rates of differential methylation

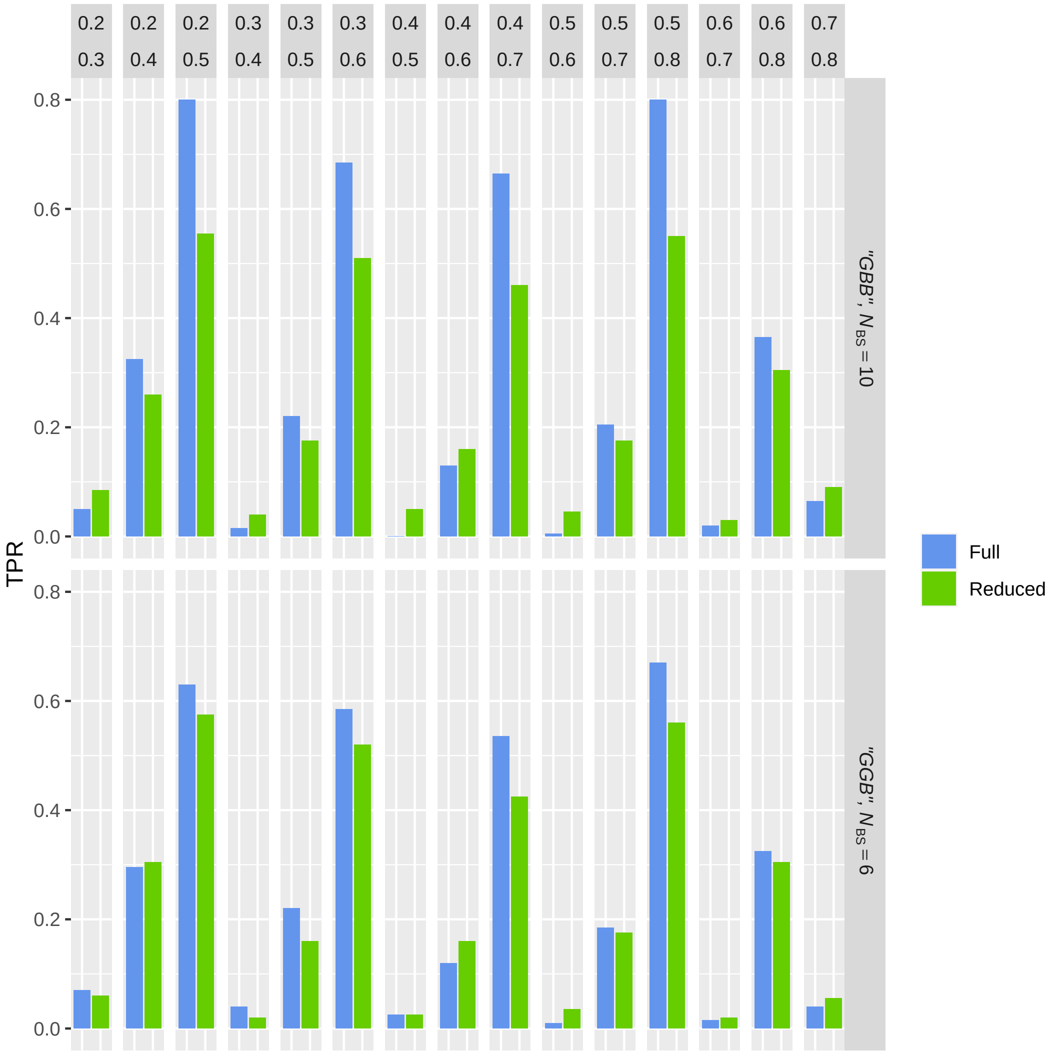

Figure S7: The barplots show true positive rates of differential methylation. True positive differential methylations were generated using samples with θ values shown on the top x-axis, e.g. $\theta_1 = 0.2$ and $\theta_2 = 0.3$ (n=200), and true negative differential methylations using samples with $\theta_1 = \theta_2$ (n=200). The cutoff used is positive differential methylation when BF > 1. Only samples using ADVI approximation were included.
